# Real-Time External Control Combined with Image Post-Processing for Mitigating SEM Vibration Distortion

**DOI:** 10.3390/mi17030315

**Published:** 2026-03-02

**Authors:** Jieping Ding, Ling’en Liu, Mingqian Song, Junxia Lu, Yuefei Zhang

**Affiliations:** 1 School of Materials Science and Engineering, Beijing University of Technology, Beijing 100124, China; 2 School of Materials Science and Engineering, Zhejiang University, Hangzhou 310058, China

**Keywords:** Scanning Electron Microscope (SEM), vibration distortion, real-time active suppression, external control, image post-processing

## Abstract

Scanning electron microscopes (SEMs) are crucial for material characterization. They are highly susceptible to vibration from environmental sources, internal components, and other external factors, which can impair measurement accuracy. Traditional solutions are limited in addressing multi-source vibrations: passive isolation struggles with internal vibrations, while image post-processing cannot fundamentally correct large-amplitude deviations in the electron beam. Therefore, this study proposes a hybrid framework that combines real-time active hardware suppression with post-processing to mitigate vibration-induced distortion in SEM images. Using a self-developed external controller and software, the framework extracts periodic vibration features via FFT, quantifies scan line horizontal offset, and implements real-time inverse offset during imaging to suppress dominant-frequency vibration at the source. An adaptive median filtering algorithm is integrated with a Laplacian edge enhancement algorithm to address residual edge burrs, thereby balancing distortion suppression and detail preservation. Experiments at 100 kx magnifications demonstrate notable correction effects: the peak-to-peak value, edge transition width (ETW), and no-reference image quality (NIQE) score are reduced by 39.4%, 91.7%, and 58.9%, respectively. Consistent correction trends are observed at 50 kx, with periodic vibration distortion essentially eliminated across both magnifications. Furthermore, distortion can be regulated through the phase interaction between dwell time and vibration period, making the strategy universally applicable and easy to implement. Without the need for vibration source localization, the framework is compatible with various types of vibration interference. It provides a solution for mitigating vibration impacts in high-magnification, precise characterization of SEMs and offers a reference for anti-vibration optimization of other microscopic techniques, such as transmission electron microscopy (TEM) and atomic force microscopy (AFM).

## 1. Introduction

Scanning Electron Microscopy (SEM) is an important method for high-resolution characterization of material microstructures, and its imaging quality directly determines the reliability of microscopic structure observation and quantitative analysis [1,2]. However, SEM image acquisition is vulnerable to vibration interference, including environmental disturbances, internal instrument vibrations (e.g., vibrations from the electron beam control module), vibrations from supporting equipment (e.g., start-stop operations of vacuum pumps and rotary pumps), and vibrations from auxiliary components in in situ experiments. These vibration interferences can cause the electron beam scanning trajectory to deviate, and the images usually exhibit periodic distortions such as serrated feature edges and stripe artifacts, seriously affecting the authenticity and measurement accuracy of the images [3,4,5].

Numerous existing studies have focused on mitigating the impact of vibrations on imaging. Traditional methods primarily rely on identifying and eliminating the vibration source [6,7], as well as utilizing passive vibration isolation [8,9]. Vibration source identification typically involves measuring vibration frequencies, matching the equipment to locate vibration sources, and optimizing the precision of instrument installation. Passive isolation blocks vibration transmission through devices such as vibration-damping platforms and rubber pads, thereby reducing most vibration interferences. However, these traditional methods struggle to accurately identify all vibration sources, especially in scenarios with multiple vibrations. Additionally, after most vibration interferences are removed, the remaining micro-vibrations still cause image distortion, and the impact of vibrations becomes more pronounced, particularly in high-magnification imaging. Image post-processing is an effective offline approach to address image distortion, with existing solutions including machine learning-based modeling [10,11,12,13] and filtering techniques [14,15,16,17]. In recent years, the application of machine learning in microscope image processing has gradually become widespread, exhibiting excellent performance in handling complex scenarios—such as image denoising and super-resolution reconstruction. Despite their outstanding performance, these advanced methods are often accompanied by high training costs and complex computations, limiting their application in real-time scenarios. Deep learning-based methods have exhibited great potential in microscopic image distortion correction, including the global wavelet-integrated residual frequency attention regularized network [18], trustworthy lightweight multi-expert wavelet transformer [19], and fused domain-cycling variational generative network [20]. These methods integrate wavelet transform and attention mechanisms, excelling in offline distortion suppression and detail enhancement via complex feature extraction. However, similar to general machine learning methods, they rely on large-scale labeled SEM datasets, suffer from high computational complexity, and cannot be deployed on hardware for real-time correction, making them unsuitable for SEM in situ experimental scenarios. Filtering methods offer a convenient way to mitigate impacts by suppressing information related to high-frequency vibrations, but a single filtering method has limited effectiveness in correcting large-amplitude vibrations, leading to severe blurring of microstructures. Spectrum analysis is a widely used classical signal processing method, commonly applied in electromagnetic interference analysis and vibration detection [21,22]. For instance, offline calculation using the discrete Fourier transform (DFT) method followed by writing into the system circuit for correction [23,24] reduces the computational burden. With the continuous exploration of electron microscope scanning strategies [25,26] and the improvement of algorithm efficiency, hardware processing methods integrating frequency analysis and phase control have made real-time active vibration suppression feasible. Different from the aforementioned passive vibration isolation methods, hardware-based real-time active vibration suppression technology can significantly weaken the impact of vibrations on imaging, providing a solid foundation for subsequent image processing.

In this study, we propose a hybrid framework combining real-time active hardware suppression and algorithm-based post-processing. This framework can flexibly and customarily solve SEM image distortion caused by multi-source vibrations. We have developed a controller and software system [27], which realizes real-time correction of vibration displacement through external control of imaging. For residual interference after real-time correction, a combined algorithm of adaptive median filtering and edge enhancement is adopted for further optimization, balancing distortion suppression and detail preservation. The objectives of this study are threefold: (1) to propose a hardware-algorithm collaborative framework that efficiently suppresses periodic vibration distortion at a high magnification of 100 kx; (2) to achieve vibration distortion correction without precise localization of vibration sources, adapting to multi-source vibration scenarios such as environment, internal instrument, and in situ loading (e.g., tension); (3) to provide a solution without modifying the SEM, offering a reliable distortion correction strategy for scenarios where passive vibration isolation or single filtering methods are ineffective.

## 2. Methodology

### 2.1. External Control Imaging

**Figure 1:**
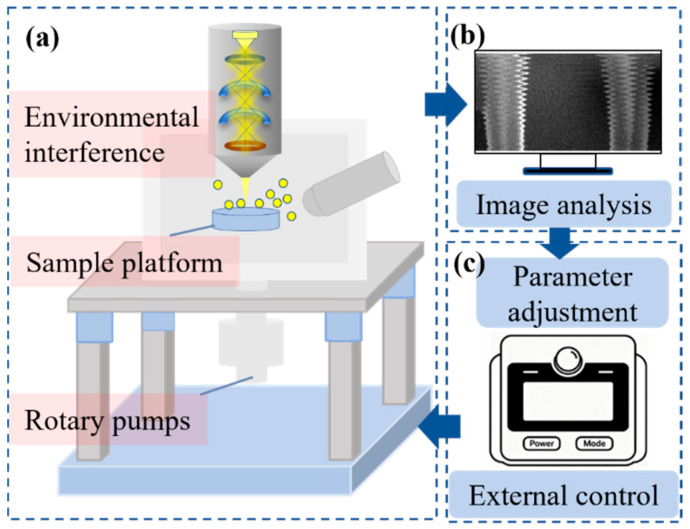
Schematic of SEM image acquisition with an external vibration control system: (**a**) SEM system setup, showing key sources of vibration interference and the sample platform; (**b**) Distorted SEM image and the subsequent image analysis step; (**c**) External control unit, used for parameter adjustment to mitigate vibration distortion.

During conventional SEM imaging, the electron beam is focused and deflected, then hits the sample point by point, and the excited electron signals are collected and converted by the detector, ultimately presenting the microscopic morphology of the sample. In this study, a self-developed external control and software system was utilized to focus on realizing dynamic regulation of the scanning trajectory and real-time adaptation of parameters. The entire external imaging process forms a complete closed loop, as shown in Figure 1. Distorted images affected by vibration are collected and the offset is calculated (Figure 1a,b), then the scanning parameters are set through the software (Figure 1c), a customized scanning signal is output to achieve dynamic correction of the scanning line, and finally the corrected image is output. This system uses a Field-Programmable Gate Array (FPGA) as the core control unit, which can precisely adjust the deflection range of the electron beam and supports on-site programming based on the vibration offset, achieving active suppression of vibration distortion. The detailed controller design can be found in Figure S1 of the supporting materials. The external controller and software system are compatible with various types of SEM (such as Tescan (Brno, Czech Republic) and Thermo Fisher Scientific (Waltham, MA, USA), etc.). The experiment was conducted on a Tescan Mira4 SEM (Brno, Czech Republic) with multiple vibration sources and vibration sources including the sample platform, the rotating pump, and other areas (the red mark in Figure 1a). The specific vibration test is shown in Figure S2.

### 2.2. Real-Time Scan Line Offset

**Figure 2:**
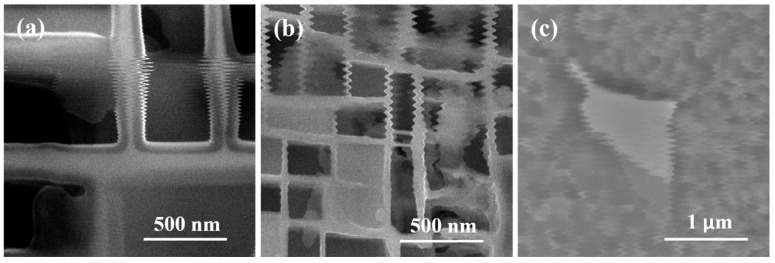
Image distortion caused by vibration: (**a**) Environmental interference; (**b**) Shaking of the sample stage during in situ experiments; (**c**) Other components.

The essence of SEM image distortion caused by vibrations is that the electron beam is disturbed by vibrations during the sample scanning process, leading to the deviation of the scanning trajectory from the preset path. This is manifested as periodic vertical misalignment between adjacent horizontal scan lines, ultimately resulting in typical features such as serrated sample edges and periodic stripes in the image. A second-generation nickel-based single-crystal superalloy was selected as the sample in the experiment, and the γ′ phase on the sample surface was slightly etched. This treatment facilitated the observation of the cubic lattice distribution of the γ′ phase and the γ/γ′ phase interface profile via SEM, thereby intuitively reflecting the offset law of scan lines. Figure 2 shows the scenarios of artificially applied environmental interference, sample stage jitter during in situ experiments, and vibrations of SEM supporting facilities, respectively.

**Figure 3:**
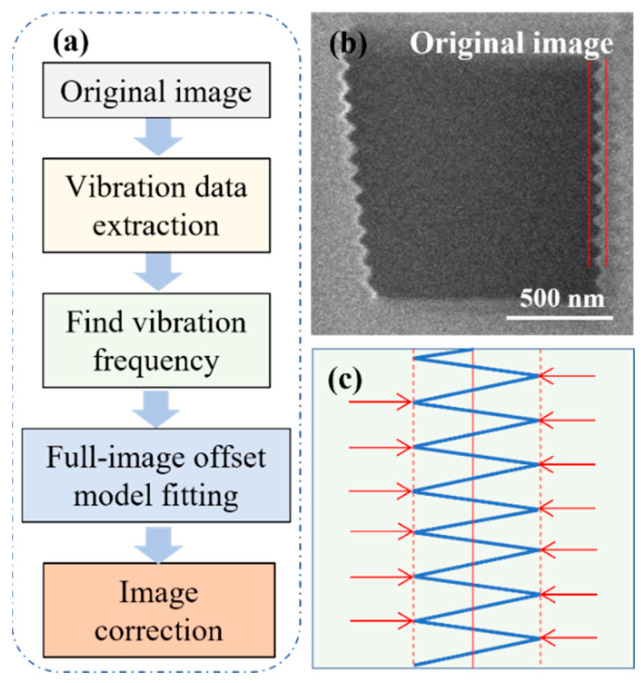
Schematic of vibration distortion correction workflow and mechanism: (**a**) Vibration correction algorithm flowchart, from image input to distortion correction output; (**b**) Vibration-distorted original SEM image: the red curve highlights sawtooth edge distortion; (**c**) Scan-line correction mechanism: blue lines represent original deviated scan paths, red arrows indicate the direction of offset correction, and red solid lines denote the corrected scan paths.

In this study, vibration distortion is equivalent to a periodic offset between horizontal scan lines. To significantly improve computational speed while ensuring frequency analysis accuracy, we propose a correction approach based on Fast Fourier Transform (FFT) (Figure 3a). This process first extracts the periodic features of distorted original images via FFT, and quantifies the horizontal offset of scan lines combined with phase analysis. Subsequently, the external controller is used to control the deflection of the electron beam, ultimately achieving full-image distortion correction.

As shown in Figure 3b, a region of interest (ROI) is selected from the vibration-distorted image by framing. This region contains continuous serrated edges to ensure effective extraction of offset signals. The size of the ROI is set to M rows × N columns, and this size parameter is recorded for subsequent calculations. The offset of scan lines causes phase changes in their grayscale distribution. Phase information is extracted via FFT [28], from which the physical offset can be inferred. The specific steps are as follows:

Firstly, a dynamic harmonic order H is introduced to constrain the frequency domain components, ensuring that the calculated offset remains within the physically reasonable range. Specifically, the vibration offset of SEM typically falls within a reasonable range from sub-pixel to several pixels. Combined with the spatial scale characteristics of the image, the value of H is set to 1/8 of the number of columns in the ROI, which concentrates the limited frequency resolution on the dominant harmonic range of the vibration signal [29]:

(1) H=max(1,round(N/8))

where round denotes the rounding function, and max(1,∙) ensures that the harmonic order is not less than 1 (to avoid frequency component invalidation).

Secondly, for each scan line y(y=1,2,…,M) within the ROI, its grayscale sequence is extracted and transformed into the frequency domain via FFT, yielding a complex-valued spectrogram $Y_{y}$. Since the index of the FFT result starts from 0 (0 corresponds to the DC component), the index of the frequency component corresponding to the $H^{th}$ harmonic is H+1. The phase $\varphi_{y}$ of this component is extracted.

(2) $$\varphi_{y}=angle(Y_{y}\left[H+1\right])$$

where the Nyquist frequency constraint is applied to ensure the physical validity of the frequency component. angle denotes the complex phase calculation function; and the condition H≤N/2 must be satisfied. Otherwise; the offset of the scan line is recorded as an invalid value (NaN). According to the spatial displacement-phase relationship of the Fourier transform; the phase change is converted into a pixel-level offset d(y), with the core formula as follows:

(3) $$d\left(y\right)=\frac{\varphi_{y}\cdot N}{2\pi \cdot H}$$

where $d\left(y\right)$ represents the horizontal offset of the $y^{th}$ scan line, N denotes the number of columns in the ROI, and 2π is the phase period. The calculated offse sequence $d={\left[d\left(1\right),d\left(2\right),\ldots ,d\left(M\right)\right]}^{T}$ undergoes validity screening to remove NaN values, resulting in a valid offset sequence $d_{valid}$, whose length is denoted as K(K≤M). The offset sequence $d_{valid}$ implies the periodic characteristics of vibrations, and a normalized autocorrelation function R(τ) is employed to analyze and extract the real period of vibrations. To eliminate the interference of DC components on periodicity detection, a detrending process is performed using the mean value $\bar{d}$ of the valid offset sequence, which concentrates the focus of autocorrelation analysis on the periodicity of fluctuations.

(4) $${d_{valid}^{\prime }=d}_{valid}-\bar{d}$$

(5) $$\bar{d}=\frac{1}{K}\sum_{i=1}^{K}d_{valid}(i)$$

(6) $$R\left(\tau \right)=\frac{1}{(K-\tau )\sigma^{2}}\sum_{i=1}^{K-\tau }d_{valid}^{\prime }(i)\cdot d_{valid}^{\prime }(i+\tau )$$

where τ(τ≥0) is the delay step, $\sigma^{2}$ is the variance of the valid offset sequence. $R\left(\tau \right)(R\left(\tau \right)\in \left[-1,1\right])$ value closer to 1 indicates a strong similarity of the sequence at delay τ.

Finally, to quantify the periodic variation law of the offset, a sinusoidal model is adopted to fit the offset sequence. The offset fitting model $D\left(y\right)$ is defined and converted into a linear regression form, and the design matrix X is constructed as follows:

(7) $$D\left(y\right)=A\cdot \sin\left(2\pi fy+\varphi \right)$$

(8) $$X=\left[\sin\left(2\pi fy\right),\cos\left(2\pi fy\right)\right]$$

where $D\left(y\right)$ represents the fitting offset, A is the vibration amplitude, f is the vibration frequency obtained from the autocorrelation analysis, φ is the initial phase, and y is the row index. As shown in Figure 3c, based on the full-image offset model $D\left(y\right)$, a reverse horizontal offset is performed for each scan line, that is, the two peaks approach the central reference line (red line), and the offset of the scan line is real-time corrected at the pixel level.

### 2.3. Image Post-Processing

The real-time offset of the scanning line has significantly reduced the vibration distortion of the dominant frequency, but there are still minor burrs remaining in the image, which affect the observation accuracy of the microstructure. To efficiently remove the remaining burrs, this paper proposes a joint image post-processing method combining adaptive median filtering for deburring and edge enhancement to maintain clarity. The core process of this method is divided into three steps: image format standardization, adaptive median filtering for deburring, and edge enhancement.

Firstly, grayscale normalization is performed on the image. For images with 8-bit/16-bit depth, min-max normalization is first applied to map the grayscale values to the range of 0–255, and then the image is converted to uint8 format to adapt to subsequent filtering and enhancement operations, thereby avoiding grayscale overflow and information distortion:

(9) $$I_{std}\left(x,y\right)=round\left(\frac{I_{in}\left(x,y\right)-min(I_{in})}{max(I_{in})-min(I_{in})}\times 255\right)$$

where $I_{in}\left(x,y\right)$ represents the pixel grayscale value of the 8-bit/16-bit input image, while $min(I_{in})$ and $max(I_{in})$ denote the minimum and maximum grayscale values of the image, respectively. This ensures that the grayscale distribution after normalization completely retains the features of the original image.

Secondly, the full-image filtering logic is adopted to avoid artifacts caused by region separation. A minimum filtering window of 3 × 3 is designed to smooth out the edges and reduce jaggedness, ensuring that the edge details are not overly smoothed.

(10) $$I_{med}\left(x,y\right)=median\left\{I_{std}\left(x-i,y-i\right)\right\}$$

Within the filtering window, the distinction between burrs and valid details is achieved through a two-stage median judgment: calculate the median $I_{med}$, minimum value $I_{min}$, and maximum value $I_{max}$ of the pixels in the current window. If $I_{min}<I_{med}<I_{max}$ is satisfied, the median is determined as valid detail; otherwise, the window size is increased (from 3 × 3 to 5 × 5 and 7 × 7), and the judgment is repeated until the median is valid or the window reaches the maximum size. For the central pixel $I_{xy}$, if $I_{min}<I_{xy}<I_{max}$ is satisfied, it is retained; otherwise, it is replaced with the valid median $I_{med}$, thereby realizing targeted removal of burrs.

After the median filtering, the image features may exhibit edge blurring. To address this issue, we adopted an edge enhancement strategy based on the Laplacian operator. This strategy is widely recognized in microscopic image processing for its effectiveness in preserving details and suppressing noise [30]. Based on this framework, we used the Laplacian operator to extract edges and performed weighted fusion to counteract the blurring caused by filtering. The core formula is defined as:

(11) $$I_{enh}\left(x,y\right)=0.9I_{med}\left(x,y\right)+0.1\times 0.5\left|\nabla^{2}I_{med}\left(x,y\right)\right|$$

where 0.9 and 0.1 represent the weights of the filtered image and the edge image, respectively, and the scaling coefficient of the Laplacian operator (0.5). The coefficients in Equation (11) (0.9, 0.1, 0.5) are derived based on the intrinsic characteristics of SEM images and theoretical criteria of signal processing: the weight of 0.9 for the filtered image follows the principle of “priority to preserving the image base” to ensure the main structural information (low-frequency components) remains dominant and undisturbed, the weight of 0.1 for the edge enhancement term matches the typical noise level of SEM images (5–10% of the grayscale dynamic range) to avoid over-enhancement artifacts while compensating for filtering-induced blurring, and the Laplacian coefficient of 0.5 adapts to the 8-bit grayscale dynamic range of SEM images by compressing the enhancement output to [−127, 127] to prevent grayscale clipping in the fused image.

Finally, grayscale clipping is performed on the enhanced image to ensure the stability of the effects of median filtering and edge enhancement, and to avoid artifacts caused by an extensive grayscale dynamic range:

(12) $$I_{final}\left(x,y\right)=clip(I_{enh}\left(x,y\right),255)$$

### 2.4. Image Quality Evaluation

This study employs three evaluation metrics to cross-validate the quality of corrected images for vibration distortion amplitude, edge sharpness, and overall visual quality. The details are elaborated as follows:

(1) To quantify the vibration-induced edge distortion, Peak-Peak [31] is defined as the difference between the maximum and minimum values of the grayscale sequence at feature edges, which directly characterizes the grayscale fluctuation range caused by vibration. This metric measures the degree of serrated distortion by capturing the variation amplitude of edge grayscale, with a smaller fluctuation range indicating a more effective vibration correction.
(2) Focusing on edge sharpness assessment, Edge Transition Width (ETW) [32] serves as a key metric that reflects edge blurriness by quantifying the grayscale transition span of feature edges. The specific calculation procedure involves three steps: extracting a grayscale sequence containing continuous vertical edges from the corrected image; then computing the 10% (G_10_) and 90% (G_90_) grayscale values corresponding to the dark and bright sides of the edge, respectively; and finally defining the distance between G_10_ and G_90_ as the ETW value. Vibration-induced electron beam shift leads to spatial misalignment of edge pixels, widening the grayscale transition zone and causing serrated or trailing blurriness. Thus, a smaller ETW value signifies a steeper grayscale transition, higher edge sharpness, and superior vibration correction performance.
(3) Different from the two local distortion-focused metrics above, Natural Image Quality Evaluator (NIQE) [33] is a no-reference assessment tool that evaluates overall visual quality. Its core mechanism lies in quantifying the naturalness of the image by analyzing how much its statistical features (e.g., local grayscale distribution, texture gradient) deviate from those of natural scenes. A lower NIQE score indicates that the image’s statistical characteristics align more closely with inherent natural scene laws, providing a global, human-perception-based verification of correction effectiveness.

## 3. Results and Discussions

### 3.1. Vibration Correction Performance

#### 3.1.1. Real-Time Scan Line Offset Effect

**Figure 4:**
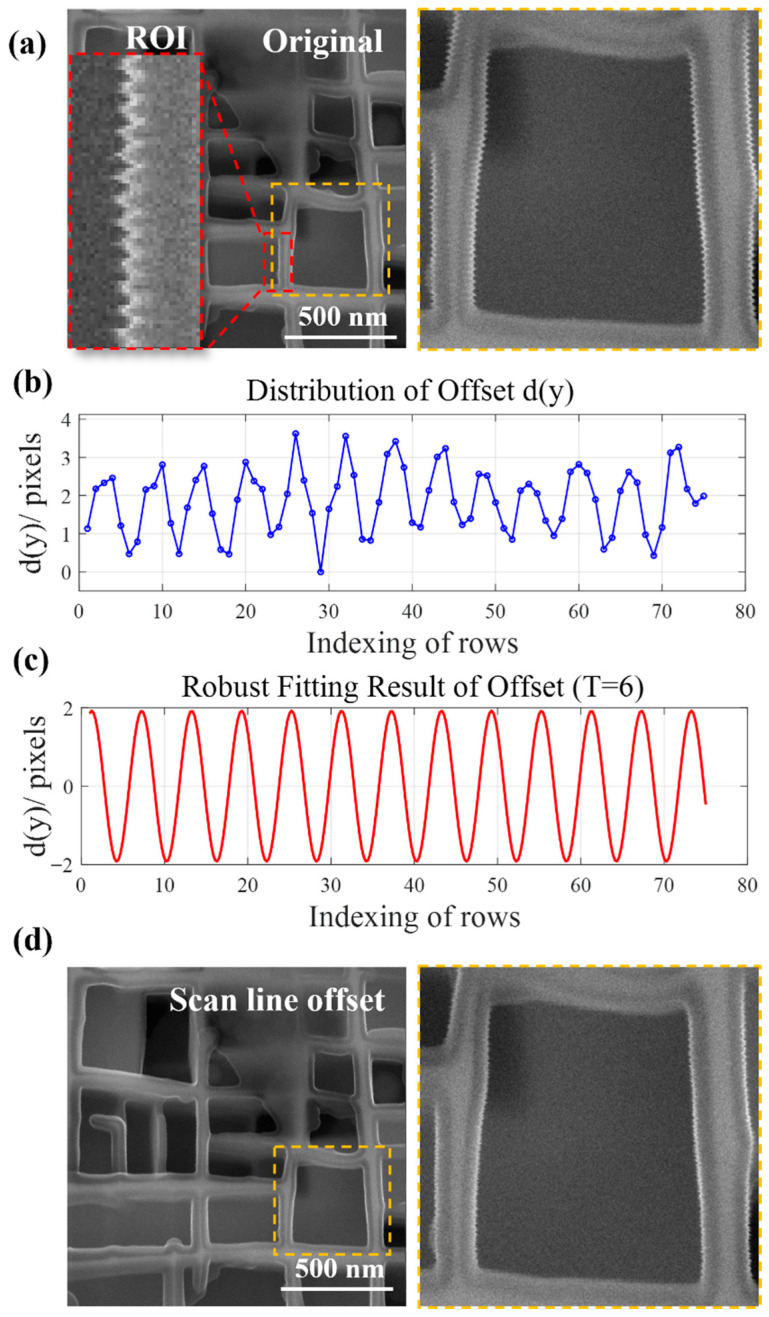
The process of image correction at a magnification of 100 kx and key quantitative results: (**a**) Original image and ROI selection; (**b**) Identification and extraction of the offset sequence of the ROI; (**c**) Fitting of the offset sequence using the sine model; (**d**) Correction of scan line offset.

Figure 4 presents the image correction workflow and key quantitative results at a magnification of 100 kx, which directly validate the real-time scan line offset correction strategy proposed in Section 2.2. Figure 4a shows the original SEM image acquired under the following experimental conditions: acceleration voltage of 15 kV, electron beam current of 3 nA, dwell time of 3.2 µs/pixel, and resolution of 1024 × 1024 pixels. An obvious periodic serrated distortion is observed at the γ/γ′ phase boundaries, as highlighted in the locally enlarged view of the distorted edges. A region of interest (ROI, 75 × 30 pixels) was selected for offset analysis—this size was determined through pre-experiments: a height of 75 rows ensures coverage of at least 10 complete vibration cycles, while a width of 30 columns guarantees the inclusion of continuous vertical edges (avoiding edge discontinuity caused by an excessively narrow width). This is crucial for the accurate extraction of offset signals. Figure 4b displays the horizontal offset sequence $d\left(y\right)$ of the ROI scan lines, calculated via FFT phase analysis (Equation (3)). The offset values fluctuate between 0 and 4 pixels, exhibiting a distinct periodic trend (period≈6 rows), confirming that the extracted offset sequence effectively reflects the actual scan line deviations induced by vibration. Figure 4c illustrates the results of Bisquare-weighted robust fitting for the offset sequence using a sinusoidal model $D\left(y\right)$ (Equation (5)). The fitting curve (red line) shows a high degree of consistency with the original offset data points, verifying that the sinusoidal model can accurately characterize the periodic variation in scan line offsets. Figure 4d presents the corrected image after applying the full-image offset model $D\left(y\right)$, where real-time offset correction of each scan line was implemented via a self-developed FPGA controller. Compared with the original image (Figure 4a), the serrated distortion at the γ/γ′ phase boundaries is significantly suppressed, directly demonstrating the effectiveness of real-time scan line offset correction at the physical level.

#### 3.1.2. Post-Processing Effects of Images

**Figure 5:**
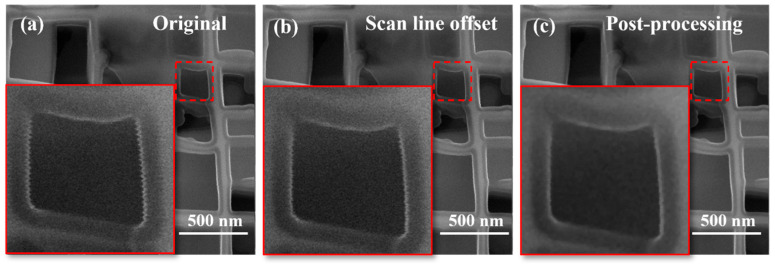
Image post-processing and comparison images: (**a**) original image; (**b**) after scan line offset; (**c**) image post-processing.

A comparison between Figure 5a,b reveals that although the image processed by scan line offset correction has significantly suppressed the dominant harmonic distortion induced by vibration, tiny residual edge burrs persist (Figure 5b). Building on the scan line offset correction, the aforementioned image post-processing method was further applied to the image. The locally enlarged view of Figure 5c intuitively demonstrates that the residual edge burrs in the post-processed image are effectively eliminated, and the structural contours are rendered smoother and clearer. This verifies the optimization effect of the proposed combined processing method on residual distortion.

#### 3.1.3. Image Quality Evaluation Results

**Figure 6:**
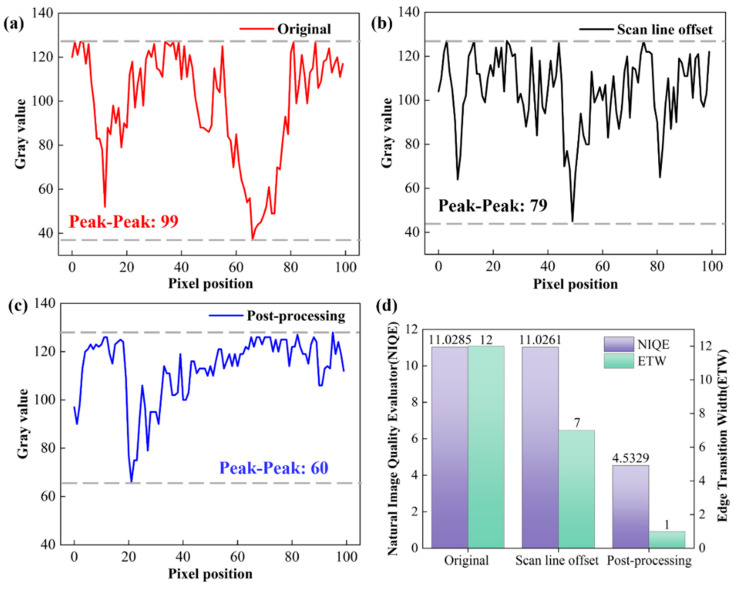
Evaluation of correction strategy using quantitative metrics: (**a**–**c**) Original image, after scan line offset, and after image post-processing; (**d**) Comparison of NIQE and ETW.

To further validate the effectiveness of the proposed correction strategy, the three aforementioned quantitative metrics were employed for comprehensive evaluation. As illustrated in Figure 6a–c, the Peak-Peak value of the original image is 99, which decreases to 79 (a reduction of 20.2%) after scan line offset correction and further drops to 60 (a reduction of 39.4%) following post-processing. This indicates that scan line offset correction can effectively compress vibration-induced grayscale fluctuations, while image post-processing further suppresses the distortion amplitude. Regarding edge sharpness (Figure 6d), the ETW of the original image is 12, which decreases to 7 (a reduction of 41.7%) after scan line offset correction. Notably, the ETW of the post-processed image plummets to 1, representing a substantial reduction of 91.7% compared to the original image. The significant decrease in ETW aligns with the variation trend of Peak-Peak, confirming that the correction strategy not only suppresses vibration distortion amplitude but also remarkably enhances edge sharpness. For overall visual quality (Figure 6d), the NIQE score of the original image is 11.0285, which remains nearly unchanged (11.0261) after scan line offset correction—indicating that scan line offset alone does not affect the perceived naturalness of the image. In contrast, the NIQE score of the post-processed image decreases to 4.5329 (a reduction of 58.9%), demonstrating that the filtering operation significantly improves overall visual quality. Collectively, the quantitative results of Peak-Peak, ETW, and NIQE comprehensively confirm the effectiveness of the proposed correction strategy from three dimensions: local distortion amplitude, edge sharpness, and global visual quality.

**Figure 7:**
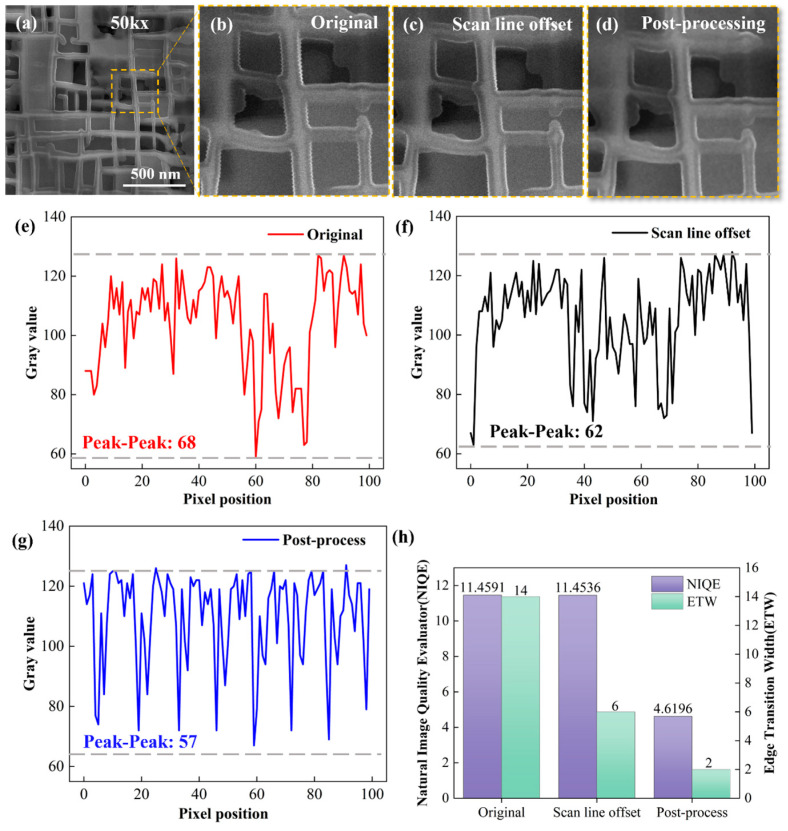
Image correction results at a 50 kx magnification and evaluation of the effectiveness of the correction strategy: (**a**–**d**) Image correction results; (**e**–**g**) Peak-to-peak ratio graphs of the original image, after scan line offset, and after image post-processing; (**h**) Comparison of NIQE and ETW.

To verify the universality of the correction strategy under different magnifications, the same quantitative evaluation was conducted on images at a magnification of 50 kx, and the results are shown in Figure 7. Figure 7a–d sequentially present the original image of the region, the image after scan line offset correction, and the final image after post-processing. An obvious periodic serrated vibration distortion can be observed at the phase boundaries of the original image (Figure 7b). After scan line offset correction (Figure 7c), the distortion is initially alleviated, and after post-processing (Figure 7d), the edges become smoother, and the contour clarity is significantly improved.

From the perspective of local grayscale fluctuation characteristics (Figure 7e–g), the Peak-Peak value of the original image is 68, which decreases to 62 after scan line offset processing (a reduction of 8.8%), and is further compressed to 57 after post-processing (a total reduction of 16.2%). This step-by-step suppression trend is completely consistent with that at 100 kx, confirming that the synergistic logic of scan line offset and post-processing is also applicable at 50 kx. The changes in quantitative indicators (Figure 7h) further support the effectiveness of the correction effect: the ETW of the original image is 14, which decreases to 6 after scan line offset correction (a reduction of 57.1%), and plummets to 2 after post-processing (a total reduction of 85.7%); the NIQE score decreases from the original 11.4591 to 4.6196 (a reduction of 59.7%).

A comparison with the results at 100 kx shows that although the initial amplitude of original distortion (Peak-Peak, ETW) at 50 kx is relatively lower, the essence is that high-magnification imaging has a more significant pixel-level magnification effect on vibration displacement. However, the correction strategy still achieves significant optimization of ETW and NIQE at this magnification, and the variation laws of the three core indicators are highly consistent with those at 100 kx. This result fully proves that the correction strategy proposed in this paper can work stably in medium and high magnification (50 kx, 100 kx) SEM characterization scenarios, and has good universality and practical value.

### 3.2. Comparison of Processing Strategies

#### 3.2.1. The Limitations of a Single Strategy

This section compares the correction effects between two single processing approaches: standalone scan line offset correction and post-processing-only methods (median filtering and mean filtering). Scan line offset correction mitigates vibration-induced displacement by real-time adjustment of the electron beam scanning trajectory, significantly reducing scan line misalignment distortion at the imaging source (Figure 6). However, as no post-processing is applied to eliminate residual minor grayscale fluctuations, the reduction in Peak-Peak value is limited (20.2%), and the NIQE score remains nearly unchanged. This indicates that standalone active suppression cannot optimize the overall natural statistical characteristics of the image.

**Figure 8:**
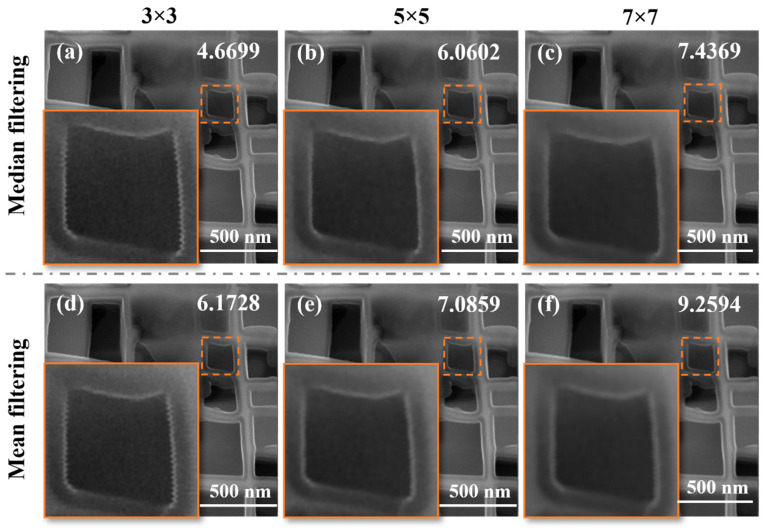
Comparison of NIQE values of the original image after direct processing using filtering methods under three filtering windows: (**a**–**c**) Median filtering; (**d**–**f**) Mean filtering.

Filtering methods such as median filtering and mean filtering mitigate grayscale fluctuations by leveraging grayscale statistics within pixel neighborhoods. As illustrated in Figure 8, a 3 × 3 median filtering window (Figure 8a) fails to effectively eliminate serrated distortion at feature edges due to its limited action range. Although 5 × 5 and 7 × 7 windows can eliminate the serrations (Figure 8b,c), the image blurriness intensifies significantly with increasing window size, and the NIQE score increases from 4.6699 to 6.0602 and further to 7.4369, respectively. Similarly, a 3 × 3 mean filtering window (Figure 8d) cannot fully eliminate serrated edges; as the window size increases (Figure 8e,f), the image blurriness further aggravates, with the NIQE score increasing from 6.1728 to 9.2594. This is because a larger filtering window exerts a stronger smoothing effect on grayscale details, leading to an increased deviation of the image from the statistical laws of natural scenes. More critically, such filtering methods can only perform post-processing on already formed grayscale distortions and cannot physically correct electron beam scanning position misalignment caused by large-amplitude vibrations (e.g., offsets induced by sample stage jitter in Figure 2b). Therefore, filtering-based methods are suitable for optimizing residual distortions from minor vibrations but cannot correct significant scanning misalignments.

In contrast, the combined strategy adopted in this study first reduces scanning misalignment from a physical perspective via scan line offset correction, suppressing large-span grayscale jumps at edges. Subsequently, the post-processing step performs refined handling of residual microscale grayscale noise. Ultimately, this approach effectively eliminates serrated edges while maintaining high image clarity. Quantitative results show that its NIQE score is only 4.5329 (Figure 6d), which is significantly lower than the results of all standalone post-processing or scan line offset correction methods. This fully verifies the synergistic advantages of the combined strategy in vibration distortion suppression and image detail preservation.

#### 3.2.2. The Advantages and Applicable Boundaries of the Hybrid Framework

The proposed hybrid framework integrating real-time scan line offset and image post-processing overcomes the drawbacks of single correction strategies, achieving a dynamic balance between distortion suppression and detail preservation. Its notable advantages depend on vibration sources with concentrated frequency distribution and distinct dominant peaks—typically from operating internal SEM components and periodic environmental equipment, whose vibrations are confined to a narrow frequency range. Notably, the framework’s performance for frequency/amplitude extraction relies on the initial ROI selection. We adopt a pre-defined ROI size, with the ROI positioned in high-contrast feature regions to isolate valid vibration signals. In addition, the reasonable range of ROI size is set to 50–100 pixels in width and 20–40 pixels in height in the method design, and minor size variations within ±10% of the 75 × 30 pixel benchmark fall within this range, with no substantial effect on extraction accuracy. In practical SEM tests, multi-source vibration is generally the superposition of narrowband vibrations rather than direct broadband or non-stationary signals, resulting in imaging distortion dominated by a primary frequency with minor components only causing slight burrs at feature edges (Figure 5b). The framework is well-suited to this characteristic, effectively mitigating distortion in multi-source narrowband scenarios, while residual burrs from minor frequencies can be reduced by expanding FFT-based extraction: screening 2–3 strong dominant frequencies via energy thresholds and adopting multi-sinusoidal model superposition for synchronous, accurate multi-frequency offset correction.

Inherent limitations arise when vibrations deviate from narrowband characteristics to broadband or non-stationary types. Broadband vibration has a continuous spectrum without distinct peaks, such as environmental noise, aging equipment interference, or sample fracture impact. FFT fails to identify valid dominant frequencies for modeling, rendering real-time offset correction ineffective, and image post-processing only smooths partial noise without eliminating root random distortion. Non-stationary vibration features time-varying frequency or amplitude (e.g., SEM start-up/shutdown frequency ramps, in situ tensile test amplitude gradients), and the sinusoidal model’s assumption of constant frequency/amplitude leads to reduced matching, increased errors, and compromised accuracy. Future work will introduce wavelet transform to replace single FFT analysis, leveraging its joint time-frequency resolution to track non-stationary frequency drift and decompose broadband multi-scale features, thereby fundamentally suppressing aperiodic fluctuation-induced distortion.

### 3.3. Phase Interaction Between Dwell Time and Vibration

**Figure 9:**
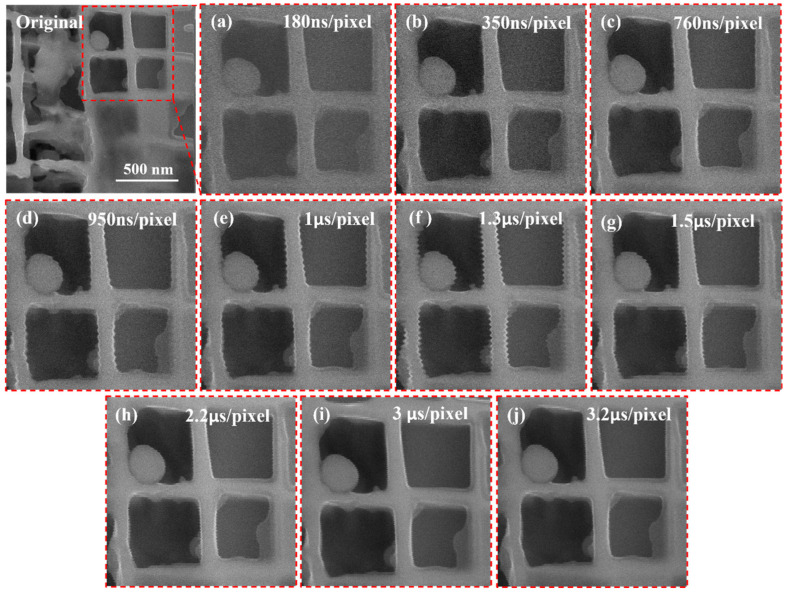
Local magnification comparison of continuously collected SEM images under ten different pixel dwell times: (**a**–**j**) Corresponding to pixel dwell times of 180 ns/pixel, 350 ns/pixel, 760 ns/pixel, 950 ns/pixel, 1 μs/pixel, 1.3 μs/pixel, 1.5 μs/pixel, 2.2 μs/pixel, 3 μs/pixel, and 3.2 μs/pixel, respectively.

To explore the regulatory mechanism of dwell time on vibration distortion, this study continuously collected images with a 100 kx magnification and a resolution of 1024 × 1024 pixels under different dwell times. We customized 10 types of dwell times ranging from short to long (180 ns/pixel to 3.2µs/pixel), with adjacent time intervals much smaller than the conventional settings of commercial SEM. From the local magnification figures in Figure 9a–j, it can be seen that the severity of vibration distortion shows a trend of first increasing and then decreasing with the extension of dwell time. The core mechanism lies in the phase superposition effect between the dwell time and the vibration period, and can be analyzed through the time accumulation law of vibration displacement:

(1) The short dwell time (180 ns/pixel) results in no significant accumulation of displacement and weak distortion (Figure 9a). When the dwell time is much shorter than the vibration period, the relative displacement change caused by vibration during the acquisition period of a single pixel can be ignored, and there is no obvious cumulative effect of displacement. At this time, the image only shows extremely slight gray-scale fluctuations and no geometric distortion that can be discerned by the naked eye, indicating that the short dwell time can weaken the interference by compressing the vibration influence window of a single acquisition.
(2) After resonance between dwell time and vibration period (such as around 1.3 μs/pixel), the phase synchronization is superimposed, and the distortion is maximized (Figure 9f). The electron beam is in the same phase interval of vibration displacement during each pixel acquisition period (such as continuous forward offset), and the displacement increment is continuously superimposed, forming “resonant distortion amplification”, manifested as the most significant sawtooth edges.
(3) The dwell time deviates from resonance (such as 3 μs/pixel), the phase alternates and cancels each other, and the cumulative effect is weakened (Figure 9j). When the dwell time is significantly longer than the vibration period, the acquisition period of a single pixel covers multiple vibration cycles (e.g., a dwell time of 3 µs/pixel can accommodate 2–3 vibration cycles with a period of 1.3 µs). At this point, the vibration displacement directions alternate during the acquisition periods of adjacent pixels (both positive and negative offsets coexist), and partial displacements cancel each other out. The overall accumulated displacement is significantly reduced, and the serrated distortion of the image is alleviated accordingly. This indicates that long dwell time can weaken periodic phase coupling through the time-averaging effect.

Notably, previous studies on dwell time optimization have mostly focused on the field of engineering vibration testing, with the core goal of avoiding equipment structural damage by evading resonance periods [34]. This study extends the correlation between dwell time and vibration period to the field of SEM imaging quality control, which boasts the advantages of simple operation and high universality. Meanwhile, it can quickly optimize imaging quality without hardware modification or complex algorithm addition, distinguishing it from the anti-phase compensation method that requires pre-measuring vibration frequency and modifying circuits [24]. Additionally, this strategy holds potential application value in in situ dynamic loading experiments: by dynamically adjusting the dwell time, it can flexibly cope with additional vibration interference caused by the installation of in situ devices, thereby reducing experimental errors. It should be noted that although shortening the dwell time can mitigate vibration distortion, it may lead to insufficient signal accumulation of individual pixels and thus sacrifice the image signal-to-noise ratio (SNR). On the contrary, excessively long dwell time will reduce imaging efficiency, making it difficult to meet the needs of dynamic observation. Therefore, future research should further divide the optimal parameter range of dwell time based on specific imaging objectives to achieve a multi-objective balance among anti-vibration distortion, SNR, and imaging efficiency.

## 4. Conclusions

This study addresses periodic vibration-induced distortions in SEM imaging via a hybrid framework combining hardware real-time active suppression and lightweight image post-processing. Key conclusions are as follows:

(1) The framework achieves stable correction: at 50 kx and 100 kx magnifications, periodic distortion is essentially eliminated—at 100 kx, peak-to-peak offset, ETW, and NIQE score are reduced by 39.4%, 91.7%, and 58.9%, respectively; corresponding reductions at 50 kx are 16.2%, 85.7%, and 59.7%, validating its adaptability to high magnification SEM characterization.
(2) A dwell time-vibration coupling law is revealed: distortion peaks when dwell time equals integer multiples of the vibration period (phase-synchronized accumulation) and weakens with deviation (phase cancellation), enabling rapid distortion mitigation via parameter regulation without hardware modification.
(3) The scheme avoids vibration source localization and suits multi-scenario vibrations, with real-time hardware correction and lightweight post-processing; however, it currently adapts only to single-dominant-frequency vibrations. Future work will optimize multi-order resonance correction via multi-frequency feature extraction and sub-pixel offset modeling.

In summary, this study presents a reliable anti-vibration scheme for high-magnification and accurate SEM characterization. It also offers a valuable reference for anti-interference optimization of other high-resolution microscopy technologies, including TEM and AFM. The framework is adaptable to both techniques but needs targeted adjustments based on their distinct distortion sources and challenges. TEM experiences vibration distortion mainly from specimen stage instability and magnetic lens misalignment. Its adaptation adopts real-time electron beam correction coupled with lens current fine-tuning. The key challenge here is matching correction latency to TEM’s ultra-high imaging speed. AFM distortion is driven by tip-sample interaction and cantilever vibration. Beam correction is therefore replaced with probe scanning path regulation for its adaptation. The main challenge is high-precision synchronization between path correction and real-time cantilever detection. These targeted adjustments form the core direction for its extension to other microscopy techniques.

## Data Availability

The original contributions presented in this study are included in the article/Supplementary Materials. Further inquiries can be directed to the corresponding authors.

## Supplementary Materials

The following supporting information can be downloaded at: https://www.mdpi.com/article/10.3390/mi17030315/s1, Figure S1: The main part of the self-developed external controller. Figure S2: Measure the frequency of the vibration source using a laser vibration meter. Video S1: Real-Time Vibration Correction.
