# Supplementary material for: Real-Time External Control Combined with Image Post-Processing for Mitigating SEM Vibration Distortion
Source: Micromachines (Basel). 2026 Mar 2;17(3):315. doi: 10.3390/mi17030315 (PMC13027915; doi:10.3390/mi17030315)
Supplement: Supplementary file 1 [file micromachines-17-00315-s001.zip › Supplementary FigS1&FigS2.pdf]

Supplement materials

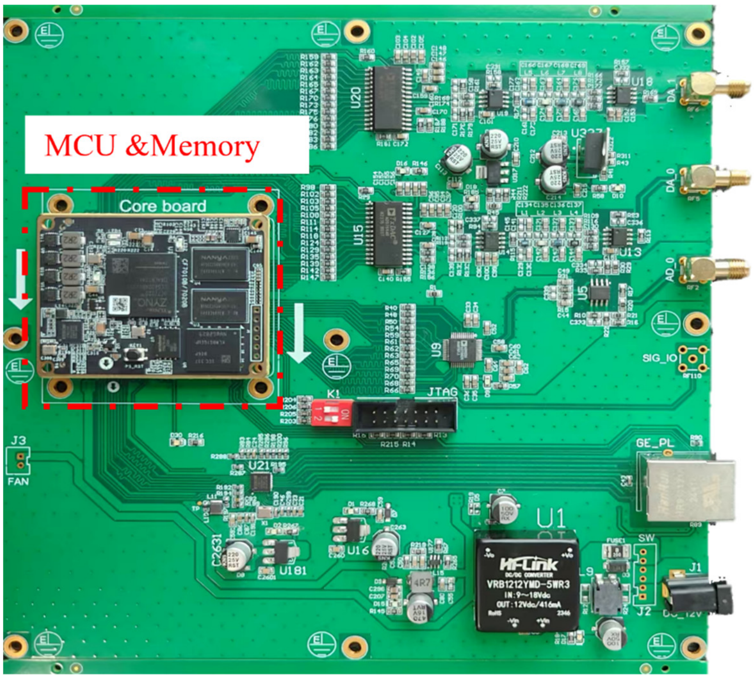

Fig.S1 The main part of the self-developed external controller.

Data processing: The data processing unit consists of an MCU and a data storage unit. The MCU utilizes the field-programmable gate array (FPGA) of the ZYNQ-7020 (Xilinx company), which integrates a dual-core Advanced RISC Machine (ARM) Cortex-A9 Multiprocessor Core (ARM Cortex-A9 MPCore) processor. The main frequency rate of ZYNQ-7020 can reach up to 667MHz, endowing it with high computational capabilities. The data storage unit employs DDR4 memory (Micron company) with a data transfer rate of up to 4266 megahertz. The speed of data processing provides support for real-time scanning line offset.

Data communication: The data communication module is also known as the upper computer interface. The Gigabit Ethernet data communication module is used to transmit the image matrix generated by the data processing unit to the host computer for image display and analysis. The data communication module adopts the YT8511C chip (Motorcomm Electronic Technology Co., Ltd), which can provide a maximum data transmission rate of 1000 Mbps.

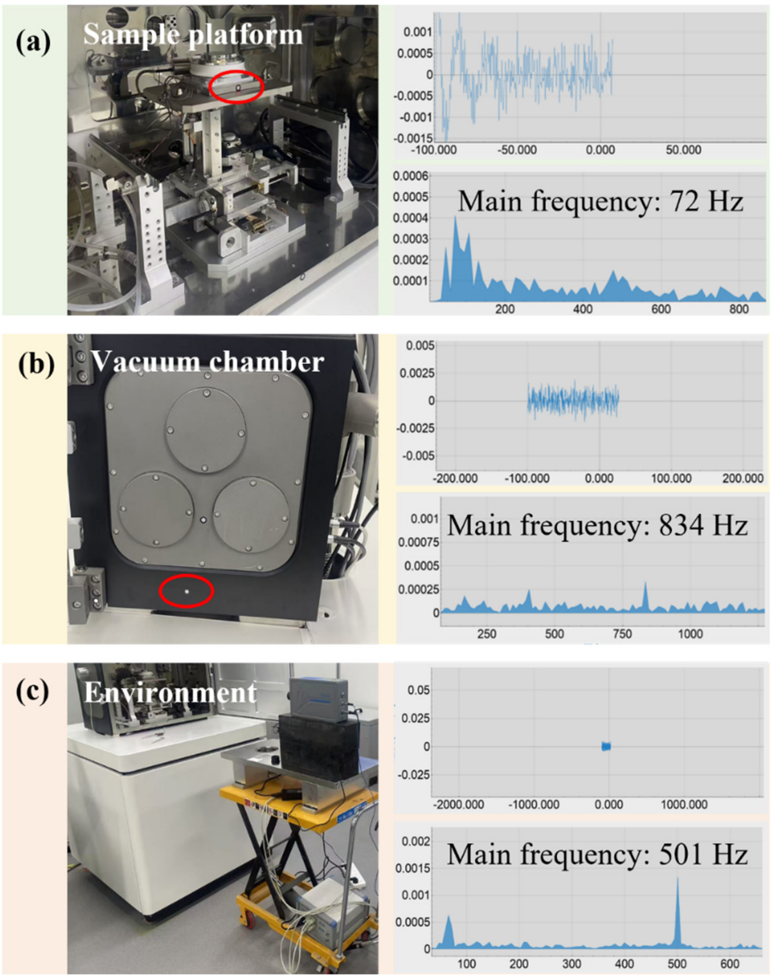

Fig.S2 Measure the frequency of the vibration source using a laser vibration meter.

The SEM in the experiment was affected by multiple vibration sources. We measured the sample platform, the chamber, and the environmental interference separately to prove that it was not a single vibration that caused the effect during the experiment.
